# Honey Glycoproteins Containing Antimicrobial Peptides, Jelleins of the Major Royal Jelly Protein 1, Are Responsible for the Cell Wall Lytic and Bactericidal Activities of Honey

**DOI:** 10.1371/journal.pone.0120238

**Published:** 2015-04-01

**Authors:** Katrina Brudzynski, Calvin Sjaarda

**Affiliations:** Drug Discovery and Development Department, Bee-Biomedicals Inc., St. Catharines, Ontario, Canada; University Hospital Schleswig-Holstein, Campus Kiel, GERMANY

## Abstract

We have recently identified the bacterial cell wall as the cellular target for honey antibacterial compounds; however, the chemical nature of these compounds remained to be elucidated. Using Concavalin A- affinity chromatography, we found that isolated glycoprotein fractions (glps), but not flow-through fractions, exhibited strong growth inhibitory and bactericidal properties. The glps possessed two distinct functionalities: (a) specific binding and agglutination of bacterial cells, but not rat erythrocytes and (b) non-specific membrane permeabilization of both bacterial cells and erythrocytes. The isolated glps induced concentration- and time-dependent changes in the cell shape of both *E*. *coli* and *B*. *subtilis* as visualized by light and SEM microscopy. The appearance of filaments and spheroplasts correlated with growth inhibition and bactericidal effects, respectively. The time-kill kinetics showed a rapid, >5-log_10_ reduction of viable cells within 15 min incubation at 1xMBC, indicating that the glps-induced damage of the cell wall was lethal. Unexpectedly, MALDI-TOF and electrospray quadrupole time of flight mass spectrometry, (ESI-Q-TOF-MS/MS) analysis of glps showed sequence identity with the Major Royal Jelly Protein 1 (MRJP1) precursor that harbors three antimicrobial peptides: Jelleins 1, 2, and 4. The presence of high-mannose structures explained the lectin-like activity of MRJP1, while the presence of Jelleins in MRJP1 may explain cell wall disruptions. Thus, the observed damages induced by the MRJP1 to the bacterial cell wall constitute the mechanism by which the antibacterial effects were produced. Antibacterial activity of MRJP1 glps directly correlated with the overall antibacterial activity of honey, suggesting that it is honey’s active principle responsible for this activity.

## Introduction

Honey antibacterial properties have been well documented along with several compounds that considerably contributed to its activity such as hydrogen peroxide [1, 2], methylglyoxal [3, 4], leptosin [5], melanoidins [6], oxidative stress and hydroxyl radicals [7, 8]. A sheer number of these compounds might suggest that honey works through a multimodal mechanism of action [9] and because of the multimodality it has remained effective in inhibiting growth of a broad spectrum of bacterial species. At present, there is not a single compound in honey with antibacterial efficacy that exceeds other contributing compounds or showing a direct correlation with the total honey antibacterial activity.

We hypothesized that honey, as a unique mixture of chemical compounds of honeybee- and nectar/pollen origins, could presumably acquire the components of an innate immune systems operating in plants and insects. In both plants and insects, mounting of innate immune responses begin with recognition and interaction with evolutionarily conserved structures on pathogens, pathogen-associated molecular patterns (PAMPs) via pattern recognition receptors (PRRs) such as Toll-like receptors (TLRs) [10, 11]. The PAMPs include bacterial cells envelope structures such as lipopolysaccharides and/or peptidoglycans [12, 13]. The interactions lead to transcriptional activation of genes coding for disease-resistance proteins in plants and antimicrobial peptides in honeybee [14, 15]. Ultimately, these pathogenesis-related gene products (disease-resistance proteins or antimicrobial peptides, respectively) are the effector molecules specialized in a direct fighting of microbial infection. Following this hypothesis, one would predict that the presence of these antimicrobial effector molecules in honey would directly influence honey antibacterial activity.

In support of this notion, our recent evidence showed the presence of a dirigent-like protein in buckwheat honey, a member of plant diseases resistance proteins [16]. Independently of this finding, we also demonstrated the existence of unknown compounds in honey that targeted cell wall (the murein/ peptidoglycan structure) in *Escherichia coli* inducing perturbations of its integrity [17]. The extent of the damage depended on honey concentrations, the exposure time and *E*. *coli* growth phase and resulted in growth inhibition and bactericidal effects. Collectively, these observations suggested a process that involved a specific recognition and interactions with the cell wall by honey compounds. In addition to the cell wall destruction, honey treatment increased permeability of outer membrane of *E*. *coli* at sub-inhibitory concentrations and the destruction of lipopolysaccharide layer (LPS) with endotoxin release at bactericidal concentrations [17]. Since the integrity of the cell wall and LPS are critical for growth and survival of bacterial cells, these cell wall-active honey compounds were of considerable interest for their possible applications as novel antibacterial agents. The need to isolate and identify the nature of the compounds directed our search towards proteins of the innate immunity. Many antibacterially active proteins, including disease resistant proteins, are glycosylated [18, 19]. Here, we used several methodologies to explore a relationship between glycosylated proteins of honey, their ability to recognize and agglutinate bacterial cells, effects they exerted on bacterial growth and survival and the mechanism underlying these effects. Finally, these antibacterial actions were corroborated with the structural identification of the glycoprotein main polypeptide by mass spectrometry.

## Materials and Methods

### Honeys

Honeys were donated by Canadian beekeepers and included unprocessed, raw samples (Table 1).

**Table 1:** Honey used and their botanical origin based on pollen analysis.

Honey	Botanical origin	Year of harvest	Melissopalynology	Donated by:
H177	buckwheat	2010	fagopyrum	Parker-Bee Apiaries
H207	rapeseed / salix	2012	brassica / salix	Podolsky Honey Farm
H208	goldenrod / rapeseed / buckwheat	2013	solidago / brassica / fagopyrum	Honey & Q Corporation
H210	wildflower	2012	rhamnaceae / ligustrum / trifolium	Bees4life

### Isolation of honey glycoproteins

Glycoproteins were isolated from 25% aqueous honey solutions using the agarose-immobilized Concavalin A spin columns (Glycoprotein Isolation Kit, ConA, Thermo Scientific, Rockford, IL, USA) according to the manufacturer’s manual. The isolation process was monitored by SDS-PAGE followed by Coomassie blue staining. In the functional assay, the flow-through fractions, wash-out fractions and fractions retained by the ConA columns were tested for their growth inhibitory activity in broth microdilution susceptibility tests.

### Determination of protein concentration

Protein quantification was performed by Bradford methods using the Bio-Rad Protein Assay (Bio-Rad Laboratories Inc. Hercules, CA, USA) according to the manufacturer instruction.

### SDS-PAGE Electrophoresis

SDS-PAGE was performed according to the method described by Laemmli, 1970 [20]. Honey proteins were analyzed by 7.5% gel separation gel with attached 5% stacking gel, while glycoproteins were analyzed on 12% gels. 30 μl of a honey solution (50% v/v) or 15μl of a fraction from ConA chromatography were mixed with the sample buffer, denatured at 100°C for 5 min and loaded on a gel. The electrophoresis was carried out in duplicate at a constant current of 100 Volts using a Mini Protean III electrophoresis cell (Bio-Rad Laboratories, Hercules, CA, USA). After electrophoresis, the gel was either stained with Brilliant Blue R-250 (Bio-Rad) for honey proteins or stained with periodic acid Schiff base (PAS-staining) to visualize glycoproteins. The molecular weight of the proteins was determined using molecular weight standards (Fermentas Life Sciences, Fisher Scientific–Canada, Ottawa, ON, Canada).

### Rapid hemagglutination assays

Rat blood collected in tubes containing EDTA was centrifuged at 3,000 × *g* for 5 min at 4°C. The pellet was washed three times in PBS containing 10 mM EDTA and then with 20 volumes of 75 mM phosphate buffer (pH 7.2) containing 75 mM NaCl. Erythrocytes were re-suspended in PBS as a 10% (v/v) suspension and immediately used in hemagglutination assay. Ten microliters of glycoprotein fractions were mixed with an equal volume of a 2% RBC suspension on glass slides. PBS and phytohemagglutinin were used as a negative and positive control, respectively. Agglutination was observed under light microscope (400x magnification) (Zeiss, Axiolab, Germany) and photographed using the digital camera and built-in software (Singer Instruments MSM 400, Somerset, UK).

### Bacterial strains and growth cultures and experimental design

Standard strains of *Bacillus subtilis* (ATCC 6633) and *Escherichia coli* (ATCC 14948) purchased from Thermo Fisher Scientific Remel Products (Lenexa, KS, USA) were grown in Mueller-Hinton Broth (MHB) (Difco Laboratories, Becton, Dickinson and Company, Franklin Lakes, NJ, USA) overnight in a shaking water bath at 37°C. Cultures were diluted with broth to the equivalent of the 0.5 McFarland standard (the turbidity of barium sulfate suspension that correspond to turbidity of ~1x10^8^ CFU/ml of bacteria).

### Broth microdilution assay and determination of the MIC

The susceptibilities of *E*. *coli* and *B*. *subtilis* (10^6^CFU/ml) to honey glycoproteins were analyzed using broth microdilution assay in a 96 well microtitre plate as described previously [1, 2]. Each well contained 100μl of inoculum and 15μl of two-fold serial dilutions of glycoproteins (starting from 40μg/ml). Bacterial growth was measured at an optical density at A_595_ nm using the Synergy HT multidetection microplate reader (Synergy HT, Bio-Tek Instruments, Winooski, VT, USA). The MIC was evaluated by recording the concentration of glycoproteins that reduced bacterial growth by 99% in comparison to a control, untreated culture, after 18 h incubation with shaking at 37ºC. Statistical analysis and dose response curves were obtained using K4 software provided by Synergy HT (Bio-Tek Instruments, Winooski, VT, USA).

### Determination of bactericidal activity of glycoproteins and the MBC

Bacterial survival, after incubation with glycoproteins, was assessed using a standard plate count. The entire contents (100μl) of experimental wells (in triplicate) from the broth microdilution assays were directly plated into the Mueller-Hinton agar (MHA) plates. The viable cells were enumerated after overnight incubation of agar plates at 37ºC and compared to the viable cell counts of control cultures. To verify the final cell density of bacteria not exposed to glycoproteins, 10μl samples from wells containing inoculum only were serially 10-fold diluted to obtain an approximate cell density of 10^4^ and 10^2^ CFU/ml and plated in duplicate, onto agar plates. The MBC was determined as the minimum concentration of glycoproteins that reduced 99% of bacterial growth.

#### Determination of time-kill kinetics and morphological changes

*E*. *coli* and *B*. *subtilis* culture (10^6^ CFU/ml in 110 μl) were grown in 96-well plates until they reached the early log phase (A_595_nm 0.2–0.3) and when each of glycoproteins (G177, G208 and G210) were added (15μl) in triplicate to separate wells at 1xMIC and 0.5x MIC concentrations. The plates were incubated at 37ºC for 18 hr. The plates served as a starting material to examine (a) the time-kill kinetics and (b) morphological changes.

### Time-kill kinetics

The time-kill kinetics were established by measuring the total viable cell counts at the time-points, 0, 15 min, 30 min, 45 min, 60 min, 120 min and 18 hr during incubation of glps with log-phase cultures. The killing curves were constructed by withdrawing 10 μl aliquots from wells containing inoculum (assay control) and experimental wells and the MBCs values were established by a standard plate count.

### Antibacterial assay of glycoproteins by agar well diffusion method

The plates were prepared using Mueller-Hinton agar (MHA) poured into layer of a 4mm deep. The 100 μl of bacterial inoculum (10^8^ CFU/ml) was uniformly spread using a bent-glass rod. Wells were performed with a sterile plastic bore of 3 mm diameter. Each well was filled with 10 μl of sample of glycosylated or deglycosylated protein (40 μg/ml) prepared in 3 dilutions; undiluted, 2x and 4x diluted with sterile water. Each plate contained well filled with ampicillin (1 μg/10 μl or 5 μg/10μl) as a positive control and BSA (5 μg/10 μl), as a negative control. In addition, the reference MHA plates were produced, inoculated with 100 μl of either *B*. *subtilis* or *E*. *coli* (10^8^ CFU/ml) that contained wells filled with series of ampicillin concentrations ranging from 2.5 to 500 μg/ml. The agar plates were incubated for 24 h at 37ºC. After incubation, the zone of inhibition of the bacterial growth was measured in mm. Tests were performed in duplicate.

### Cell morphology examination by light microscopy

The morphological changes in bacterial cells induced by glycoproteins at different growth phases were examined on glass slides using 10ul samples removed from the experimental and control wells at the time intervals described above. Images were viewed at 400 x magnifications under light microscope (Zeiss, Axiolab, Germany) and photographed using the digital camera and built-in software (Singer Instruments MSM 400, Somerset, UK).

### Bacterial agglutination assay

For qualitative determination of agglutinating activity, the 10μl of undiluted overnight cultures of *B*. *subtilis* and *E*. *coli* were mixed with 10μl glycoproteins on glass slides, while 10μl of Concavalin A (100ug/ml) and PBS were used as a positive and negative control, respectively. The mixtures were incubated for 15 min and the agglutination was inspected at 400-times magnification under the light microscope (Zeiss, Axiolab, Germany). Images were photographed using the digital camera and built-in software (Singer Instruments MSM 400, Somerset, UK).

### Scanning Electron Microscope (SEM)

For SEM analysis, overnight cultures of *B*. *subtilis* and *E*. *coli* were incubated with glycoproteins at 1xMBC for 1 hr at 37ºC. Cells were fixed in 2.5% glutaraldehyde (EM grade, Sigma-Aldrich, St. Louis, MO, USA) in 0.1 M Tris-buffered saline (TBS), pH 7.3 for 15min at room temperature. The fixed samples were dehydrated with graded ethanol series, and re-suspended in TBS. Samples (50 μl) were placed on a poly-lysine coated coverslips, air dried and dehydrated in a series of graded ethanol (70%, 95% and 100%, 2 x 2 min), dried at critical point and sputter coated with gold. The microscopic analysis was performed with a Hitachi S-530 SEM (Hitachi, Japan) operating at 20kV. Images were captured by using the Quartz PCI version 8 software.

### Enzymatic deglycosylation

Lyophilized glycoproteins (100μg) were dissolved in 30μl deionized water, heated to 100ºC for 5 min, cooled to room temperature and deglycosylated using the Enzymatic Protein Deglycosylation Kit (Sigma-Aldrich, St. Louis, MO, USA), according to the manufacturer’s instruction.

### Protein Identification by MALDI-ToF Mass Spectrometry (MS)

The deglycosylated 61 kDa protein bands of G177, G208 and G210 and 58.5 kDa protein band of G207 were excised from Coomassie blue-stained gels were processed as described in Protocols, the Biological Mass Spectrometry Laboratory, UWO (http://www.bmsl.uwo.ca/in-gel_digestion.html). Prior to mass spectrometric analysis, dried peptide samples were re-dissolved in a 0.2% formic acid and 3μl of volumes were injected into mass spectrometer.

### ESI-Q-TOF-MS/MS analyses

ESI-Q-TOF- MS/MS analyses (electrospray ionization quadrupole time of flight mass spectrometry) were performed using a Q-TOF Global mass spectrometer (Waters, Waters Corporation Milford, MA, USA) and run in positive mode using electrospray interface. MS acquisition was obtained with MassLynx 4.1 software (Waters). The columns consisted of Waters nanoAcquity UPLC column (BEH130 C18) with a trap column (Symmetry C18). Peptides were resolved at 300nl/min over a 75 min in a gradient of 0.2% formic acid (A) and methanol (B) (Table 2).

**Table 2:** ESI-Q-TOF-MS/MS chromatographic conditions.

% B	% A	Time
(methanol)	(0.2% formic acid)	(min)
5	95	0
60	40	40
95	5	42.5

All MS/MS spectra were analyzed using MASCOT search engine (http://www.matrixscience.com) against the NCBInr protein and Swiss-Prot/TrEMBL databases using peptide mass fingerprinting (PMF). The parameters used for database search were: 1) taxonomy group 2) mass tolerance of 0.2 Da, 3) one missed tryptic cleavage allowed, 4) carboamidomethylation of cysteine (as a fixed modification) and 5) oxidation of methionine (as a variable modification). Proteins were identified by MASCOT using the probability-based MOWSE score, equal to-10XLog(P), where P is the probability that the observed match is a random event. Protein scores of >53 were considered statistically significant (P<0.05) under the selected variables. PEAKS/PEAKS have also integrated PTM and mutation characterization through automatic peptide sequence tag based searching (SPIDER) and PTM identification.

### Statistical Analysis

Analyses were performed using GraphPad Instat version 3.05. (GraphPad Software Inc. La Jolla, CA, USA). Data were analyzed using a one-way ANOVA with subsequent Tukey-Kramer Multiple Comparison test or an unpaired t-test. Differences between means were considered to be significant at p<0.05.

### Ethics Statement

Blood samples were taken during rat’s euthanasia from the project AUP# 13–08–03 approved by the Brock Animal Care and Use Committee and were carried out in accordance with the Guide for the Care and Use of Laboratory Animals [21].

## Results

### Isolation of lectin-like glycoproteins from honey

We employed Concavalin A (ConA) chromatography to isolate glycoproteins, based upon the premise that many proteins of innate immunity are glycosylated [18, 19]. ConA is a lectin that specifically binds high-mannose-type N-glycans, a type of glycans often present in the cell wall of Gram- positive and Gram-negative bacteria [22]. A specific interaction of ConA with α-D-mannopyranosyl, α-D-glucopyranosyl residues of glycoproteins [23] allowed separation of glycoproteins bearing such structures from a bulk of honey proteins. Further protein purification was achieved by separating high-mannose from low-mannose glycoproteins under stringent eluting conditions (elution with 0.1 *M* methyl α-D-glucopyranoside) in order to release glycoproteins with multiple mannose groups that bound to ConA with much higher affinity [24].

**Fig 1:**
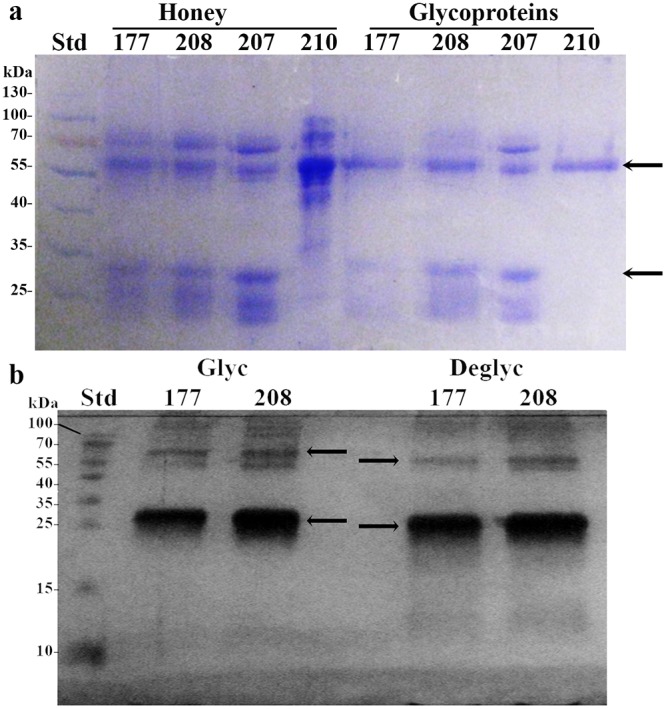
Comparison of protein and glycoprotein profiles in different honeys by SDS-PAGE. (a) Comparison of protein and glycoprotein profiles before and after ConA chromatography in different honeys. Arrows indicate positions of the 61kDa and 29 kDa proteins (b) Mobility shift of deglycosylated glycoproteins (the arrows).Std: standard molecular weight proteins.

**Fig 2:**
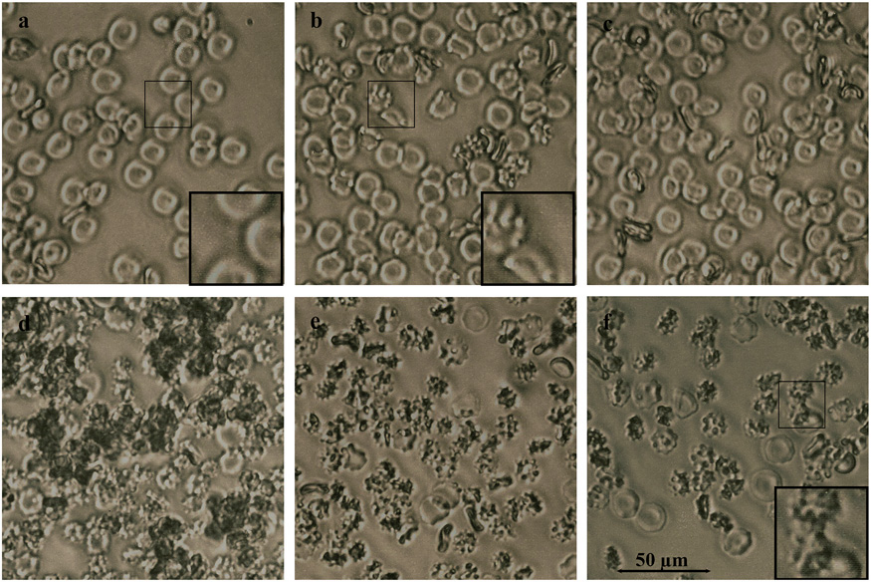
Membrane effects of honey glycoproteins on rat RBCs. Microscopic visualization of morphological changes in RBCs treated with glycoproteins or phytohemagglutinin (PHA) as compared to untreated cells. a. Control RBCs, b. RBCs treated with glp G207, c. RBCs treated with glp G210, d. RBCs treated with PHA, e. RBCs treated with glp G177 and f, RBCs treated with glp G208. Insets illustrate a transition of RBCs morphology from biconcave (a) to echinocyte form (b and f) after treatment with glycoproteins.

Among ConA- captured proteins were two abundant polypeptides; the 61 kDa protein present in all tested honeys (with exception of H207) and the 29 kDa, present in H177 and H208 (Fig. 1a). Honey H207 was enriched with mannosylated 58.5 kDa protein and 27 kDa peptide. While glycosylation increases structural stability of proteins and their resistance to proteolysis, carbohydrates in glps also function as recognition determinants of other glycoconjugates and are often responsible for cell adhesion and agglutination. To test whether honey glps have agglutinating properties, we used a rapid, direct hemagglutination assay on microscope slides using freshly isolated rat red blood cells (RBCs). The effects of the interactions between glps G177, G208, G207 and G210 with RBCs were examined under light microscope using phytohemagglutinin (PHA) as a positive control, known for the RBC agglutinating activity. PHA, another high-mannose-type lectin, clearly formed clumps of rat RBCs on microscopic slides during 30 min incubation at room temperature (Fig. 2d). In contrast, honey glps were unable to bind and agglutinate RBC under the same conditions (Fig. 2). However, the exposure of RBCs to glps G177 and G208 resulted in a rapid transition from biconcave shape of normal erythrocytes into crenated, shrank echinocytes (Fig. 2, e and f). The transition was accompanied by the formation of membranes protrusions (blebs) (Fig. 2, insets) indicating that the integrity of erythrocyte membranes became compromised. Although glps G207 and G210 did not produce such extensive membrane defects, they too influenced the changes in membrane permeability that resembled the early phases of echinocyte development (Fig. 2b). Differences in the glps profiles, as seen in the SDS-PAGE (Fig. 1), seemed to be a factor that distinguished glps actions based on severity of the membrane effects they caused to the rat RBC (Fig. 2). It could be concluded that some of these lower molecular weight polypeptides, such as those present in glps G177 and G208, contributed more to membrane disruptions than others (G207 and G210). The mass spectrometric analysis revealed that the 29kDa protein of G177 and G208 was a fragment of the 61 kDa protein while the 58.5 kDa protein and its 27 kDa polypeptide of G207 were not related to 61kDa protein and represented a different molecule (see below MALDI-TOF results).

### Bacterial agglutination, cell shape changes and cell lysis induced by honey glycoproteins

**Fig 3:**
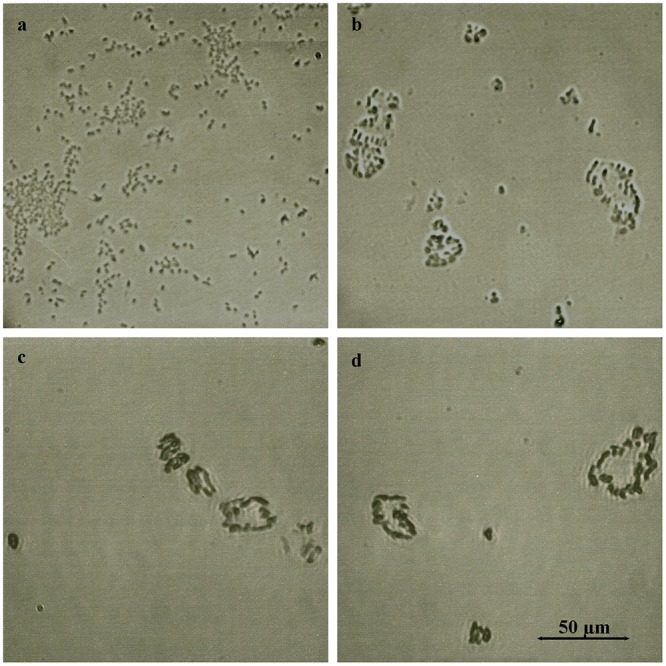
Agglutination of bacterial cells by honey glycoproteins. a. *E*. *coli* agglutination by soybean lectin b. *E*. *coli* agglutination by glp G208, c. *B*. *subtilis* agglutination by Concavalin A, and d. *B*. *subtilis* agglutination by glp G177.

**Fig 4:**
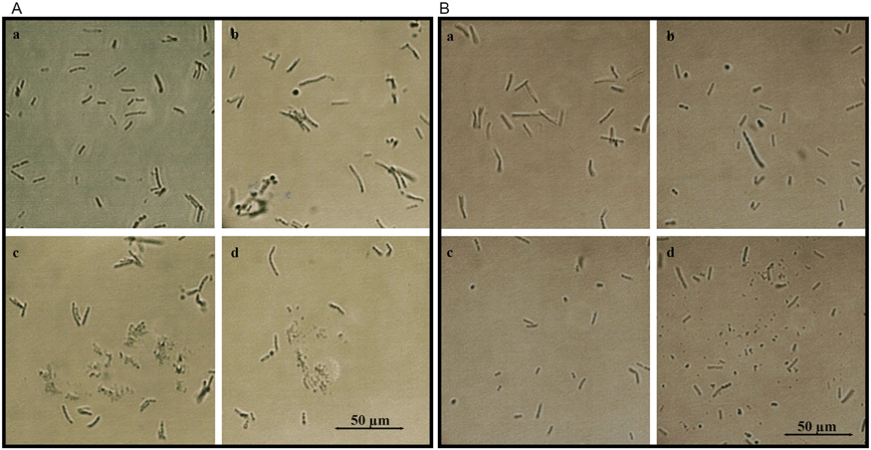
Glycoprotein- induced cell wall damage and cell shape changes in log-phase *E. coli* and *B. subtilis* cultures. A. *E. coli*. a. untreated control, b. *E. coli* incubated with G177, c. G208 and d. G210. B. *B. subtilis*. a. untreated control, b. *B. subtilis* incubated with G177, c. G208 and d. G210.

**Fig 5:**
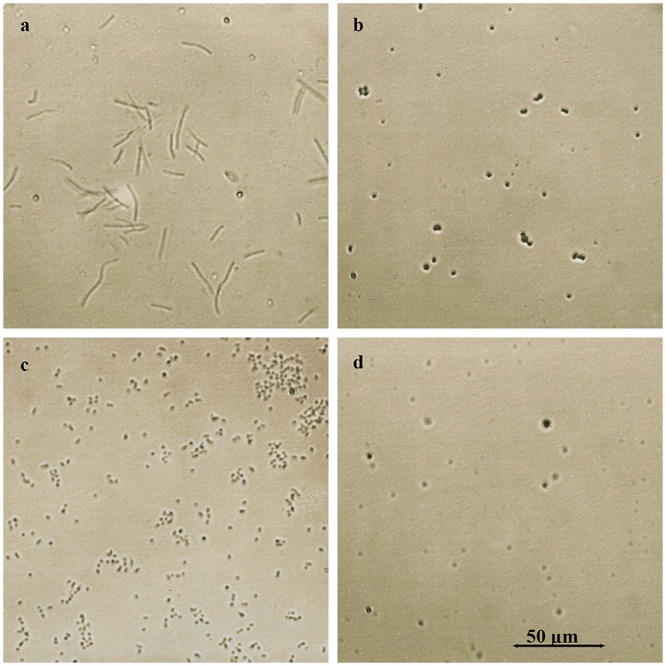
Glycoprotein- induced cell wall damage and cell shape changes in lag-phase *E. coli* and *B. subtilis* cultures. a. *B. subtilis*- control, untreated cells, b. *B. subtilis* spheroplasts formed by treatment with glp G208, c. *E. coli*–control, untreated cells, and d. *E*. *coli* spheroplasts formed by treatment with glp G208.

Exposure of overnight bacterial cultures to glps resulted in visible agglutination of both Gram- positive *Bacillus subtilis* (ATCC-6633) and Gram-negative *Escherichia coli* (ATCC-14948) (Fig. 3). All four glycoproteins clumped bacterial cells in a similar way to ConA-lectin as compared to soybean lectin (Fig. 3a and b). To investigate whether, in addition to agglutinating activity, glps have capacity to influence the growth and bacterial survival, overnight cultures of *B*. *subtilis* and *E*. *coli* (diluted to 10^6^ CFU/ml) were incubated with serially two-fold diluted glps for 18 h. At two time-points of bacterial growth, at the beginning of log-phase and at the stationary phase, 10μl aliquots were withdrawn from incubation wells for microscopic observations. The microscopic images revealed marked morphological changes that included filamentation, cell wall lysis and formations of spheroplasts (Figs. 4 and 5). The extensive disruption of the bacterial cell walls occurred indiscriminately in both Gram-positive *B*. *subtilis* and Gram-negative and *E*. *coli* (Fig. 4A and B). As a result, at the entry to a stationary phase, the majority of *E*. *coli* and *B*. *subtilis* cells had their cell-shape changed from the rod-like forms to spheroidal forms of different sizes (Fig. 5).

**Fig 6:**
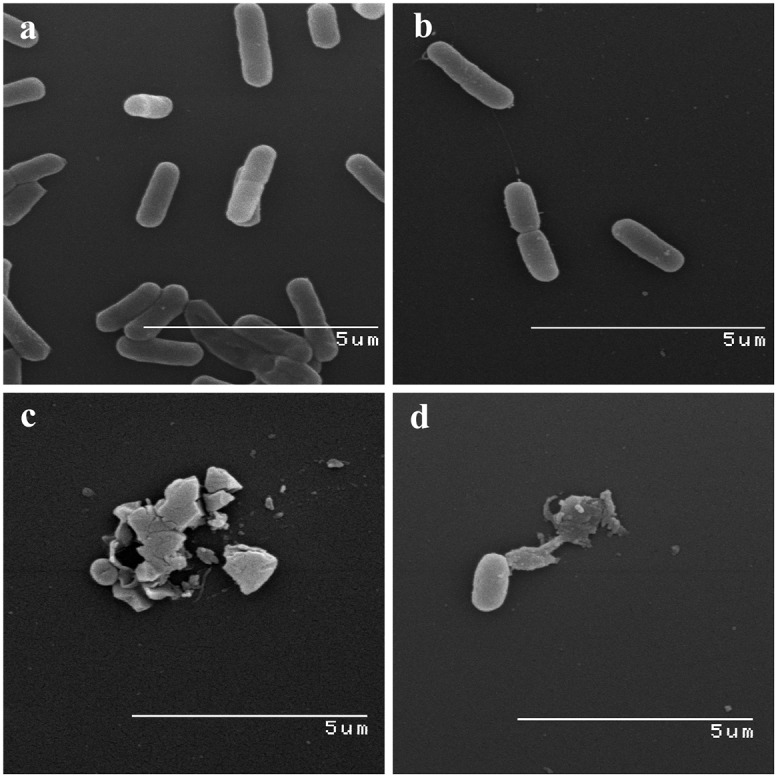
SEM images of glycoprotein-induced morphological changes in log-phase *E*. *coli* and *B*. *subtilis*. a. Control culture of *E*. *coli*, b. control cultures of *B*. *subtilis*, c. *E*. *coli* treated with bactericidal concentrations of glps, G208, d. *B*. *subtilis* treated with bactericidal concentrations of glps, G208.

The SEM images provided direct evidence that glps were involved in structural damages to the cell wall (Fig. 6). Treatment of *E*. *coli* and *B*. *subtilis* cells with bactericidal concentrations of glps led to spheroplast formation and their subsequent lysis (Fig. 6c and d). These glycoprotein-induced cell wall disruptions proved to be lethal as we demonstrated in the next paragraph.

### Growth inhibition and time-kill kinetics

Antibacterial potencies of glps were dependent on the growth phase of bacterial culture and the ratio of glps concentration to the inoculum size. Exposure of lag phase *E*. *coli* and *B*. *subtilis* cultures (10^6^ CFU/ml) to serially two-fold diluted glps ranging from 100μg/ml to 1.25μg/ml caused a complete growth inhibition. In the lag phase, there was no difference in median MIC and MBC values for both *E*. *coli* and *B*. *subtilis* obtained in three separate experiments conducted in triplicate. The MIC = MBC values varied from 13.8μg/ml (G208), 4.9μg/ml (210) and 2.5μg/ml (G177) against *E*. *coli* to 13.8–1.24μg/ml against *B*. *subtilis* (Table 3).

**Table 3:** MIC and MBC values of glycoproteins.

	Protein concentration (μg/ml)	
MIC = MBC	*E*. *coli*	*B*. *subtilis*
G177	2.5	1.24
G208	13.8	13.8
G210	4.9	2.5

No surviving cells were found using the standard plate count by seeding undiluted 100μl aliquots from the wells after 18hr incubation. The results of these functional assays together with microscopic observations indicated that the growth-inhibitory and potent bactericidal activities of glps on bacteria cells at the lag phase were likely due to their damaging action on cell membranes from which cells could not recover (see Fig. 6).

**Fig 7:**
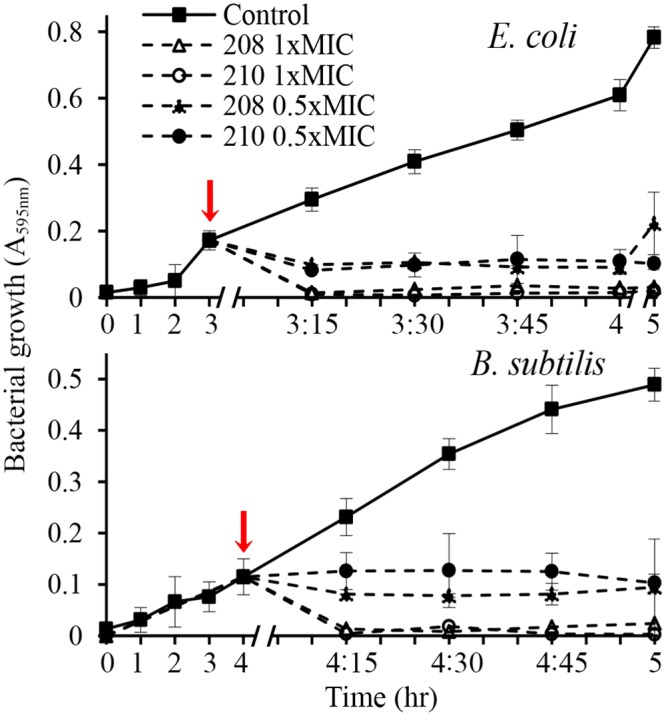
Effect of glycoprotein concentrations (1xMIC and 0.5xMIC) on growth rates of *E*. *coli* and *B*. *subtilis*. Arrows indicate time of glps application to bacterial cultures.

**Fig 8:**
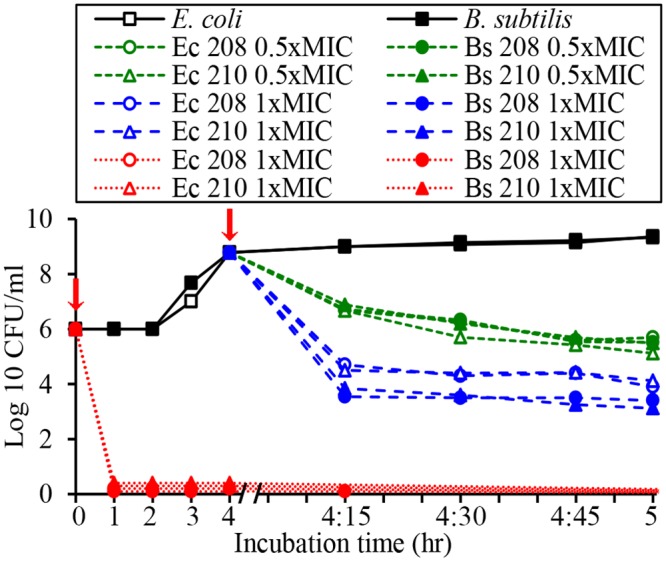
Effect of glycoprotein concentrations (1xMIC and 0.5xMIC) on survival of *E*. *coli* and *B*. *subtilis*. Arrows indicate time of addition of glps into bacterial cultures in lag phase (red line) and log phase (black line).

In contrast to lag-phase, the log phase cultures of *E*. *coli* and *B*. *subtilis* (A_595_nm 0.2–0.3, ~10^8^–10^9^ CFU/ml) showed less sensitivity to antibacterial activities of glps (Figs. 7 and 8). Although the glps application at 1xMIC resulted in a rapid reduction of bacterial growth and a >5-log_10_ reduction of viable bacteria within the first 15 min, some cells clearly survived (Fig. 8). Nevertheless, the kinetic curves generated every 15 min during 2 hr incubation showed that killing occurred during one generation time (Figs. 7 and 8). This suggested that the structural damages induced by glps to the cell wall were irreparable, leading to cell death. The longer incubation times did not change the levels of inhibition or killing rates. In contrast, the incubation of bacterial cultures with glycoproteins at 0.5xMICs reduced the growth only by about 50% during the first 15 minutes, although after that time, a steady decrease in growth rates was observed, finally reaching 80% after 1 hr (Fig. 8). Glycoproteins at 0.5xMIC were also less efficient in reducing the number of viable cells at shorter incubation times, although >3-log_10_ decrease in CFU/ml was achieved after 1 hr incubation (Fig. 8). Thus, the growth-phase and the ratio of glps concentrations to inoculum size were critical factors to produce maximal bactericidal effects.

The microscopic observations performed simultaneously with the functional assays revealed time-and dose-dependent cell shape changes. The appearance of these new phenotypes, filaments and spheroplasts (Figs. 4–6) correlated with a rapid cessation of growth and bacterial killing, respectively (Figs. 7 and 8). Therefore, the antibacterial effects of glps were related to their ability to cause structural damage to the cell wall, changing cell morphology and viability.

### Specificity of the interaction of glycoproteins with bacterial targets

**Fig 9:**
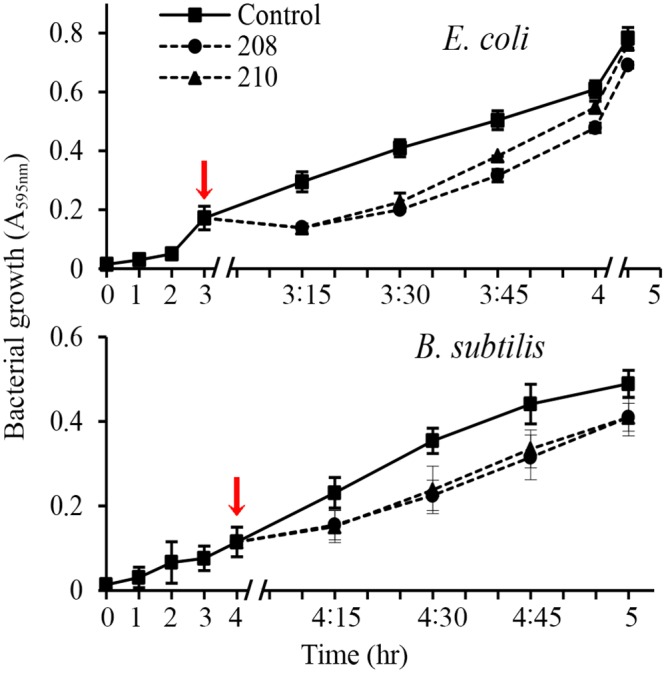
Growth inhibition of *E*. *coli* and *B*. *subtilis* by flow-through fractions from ConA chromatography. Arrows indicate time of addition of flow-through fractions to bacterial cultures.

**Fig 10:**
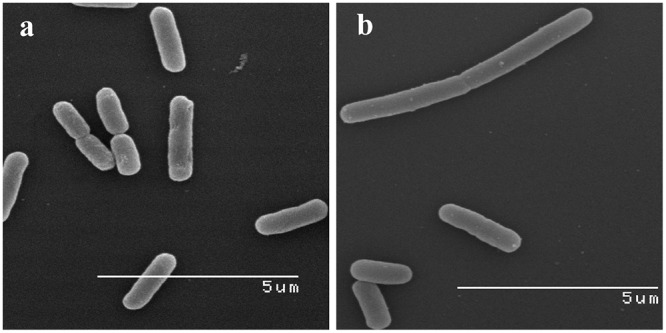
SEM images of *E*. *coli* and *B*. *subtilis* treated with flow-through fractions from ConA chromatography. Note lack of effects of flow-through fractions on bacterial cell shape.

Only glps that were captured by ConA affinity chromatography showed growth inhibitory and killing activities. The flow-through and post-wash fractions from ConA chromatography showed either greatly reduced growth inhibitory activity or were completely inactive, respectively (Fig. 9). Although the flow-through fractions showed residual bacteriostatic activity, they neither reduced the number of viable cells measured by the standard plate count nor influence bacterial cell shape (Fig. 10).

At least two interacting mannose residues must be present on protein molecule in order to bind to ConA column. Honey glycoproteins that remained bound to ConA column after extensive washings must have multiple mannose groups. It became apparent that high-mannose type N-glycans in glps participated in targeting of bacterial cell and their follow up destruction.

### Effects of protein deglycosylation on antibacterial activity

**Fig 11:**
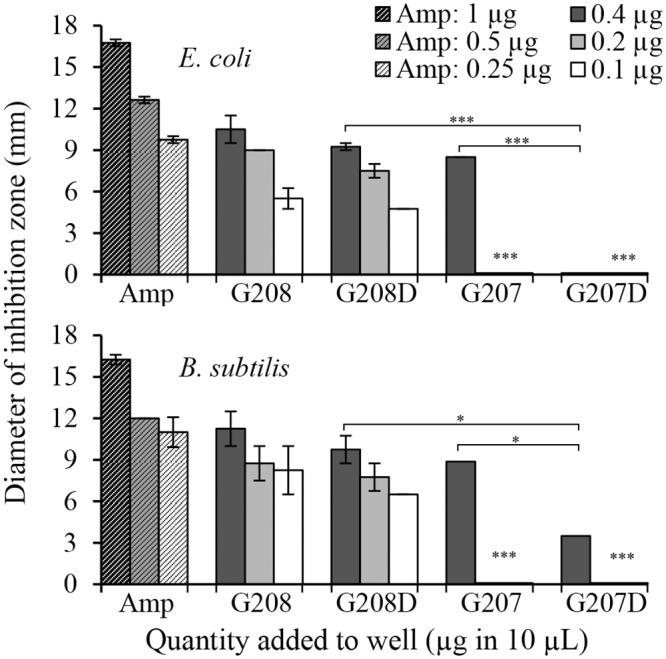
Comparison of antibacterial activities of glycosylated versus deglycosylated glycoproteins G207 and G208 analyzed using agar well diffusion assay. Inhibition zone was determined by measuring clear zone produced by test samples against *E*. *coli* and *B*. *subtilis*. Ampicillin was used as a positive control. Columns represent mean ± SEM; p<0.001 (***); p<0.05 (*).

Since honey glps displayed both agglutinating and bactericidal activity, the role of glycosylation on these activities had to be defined. Some honey glycoproteins retained by ConA column possessed only agglutinating activity, like G207, and other had both agglutinating and bactericidal activity such as G177, G208 and G210. To analyze the role of glycans in antibacterial activity, the G207 and G208 glps were subjected to 24-hr enzymatic deglycosylation using a multi-enzyme deglycosylation system, E-DEGLY (Sigma-Aldrich) that removes all *N*-linked and *O*-linked carbohydrates from glycoproteins. The glycosylated and deglycosylated glps samples (40µg/ml) were diluted two- and four-fold, and the samples’ antibacterial activities were determined using the agar well diffusion assay (Fig. 11). Ampicillin (25μg/ml to 500 μg/ml) was used as a control in these assays (Fig. 11). Despite the fact that the 24-hr deglycosylation reduced the molecular size of the main protein bands of G207 and G208 only by 10% on SDS-PAGE (data not shown), it produced significant changes in antibacterial activities of these two glycoproteins (Fig. 11). The size of the inhibition zones produced by G208 was dependent on concentrations of glps added to the well. Deglycosylation of G208 caused a reduction of the inhibition zones at all three dilutions in comparison to glycosylated G208, although these decreases were not statistically significant (Fig. 11). In contrast, G207 produced a zone of inhibition only at its highest concentration, and deglycosylation of G207 resulted in a significant decrease (*B*. *subtilis*) or a complete loss (*E*. *coli*) of the antibacterial activity of this glycoprotein.

These results established that G207 displayed mostly agglutinating activity for which glycosylation was essential. In contrast, the mechanism by which G208 produced antibacterial effect was not solely glycosylation-dependent.

### Sequencing of 61Da glycoprotein

The similar levels of cell wall lytic and bactericidal activities of glycoprotein fractions G177, G208 and G210 suggested that they all must possess the effector molecule responsible for these cytotoxic actions. The comparison of the SDS profiles of glps pointed out to a 61 kDa protein as a putative candidate because this protein was shared by all bactericidal glps. To enable identification of the 61 kDa protein, glps were extensively deglycosylated. After deglycosylation, the 61 kDa and 29 kDa bands from G208, G177 and G210 with changed electrophoretic mobilities (Fig. 1b) were excised from the Coomassie Blue stained gels, subjected to in-gel trypsin digestion followed by MALDI-TOF MS and electrospray quadrupole time of flight mass spectrometry, ESI-Q-TOF- MS/MS. Subsequently, the characteristic peptide mass fingerprints of the 61 kDa proteins were analyzed by databased search using Mascot and PEAKS, respectively. Both Mascot and PEAKS searches revealed identity of the 61 kDa protein with the major royal jelly protein 1 precursor (*Apis mellifera*, accession number: gi 58585098) with the score of 136 (greater than 95% confidence) and 298 (greater than 99.2% confidence identification), respectively (Table 4).

**Table 4:** Summary of ESI-Q-TOF- MS/MS statistical data for 61 kDa glycoproteins G208 and G210.

Protein	Accession	Score (%)	-10lgP	Coverage (%)	#Peptides	#Unique	Mass kDa	Name
G210	gi|58585098	99.2	142.9	48	20	14	48.88	MRJP-1 (56kDa)
G208	gi|58585098	99.2	140.23	40	13	9	48.88	MRJP-1 56kDa)

**Fig 12:**
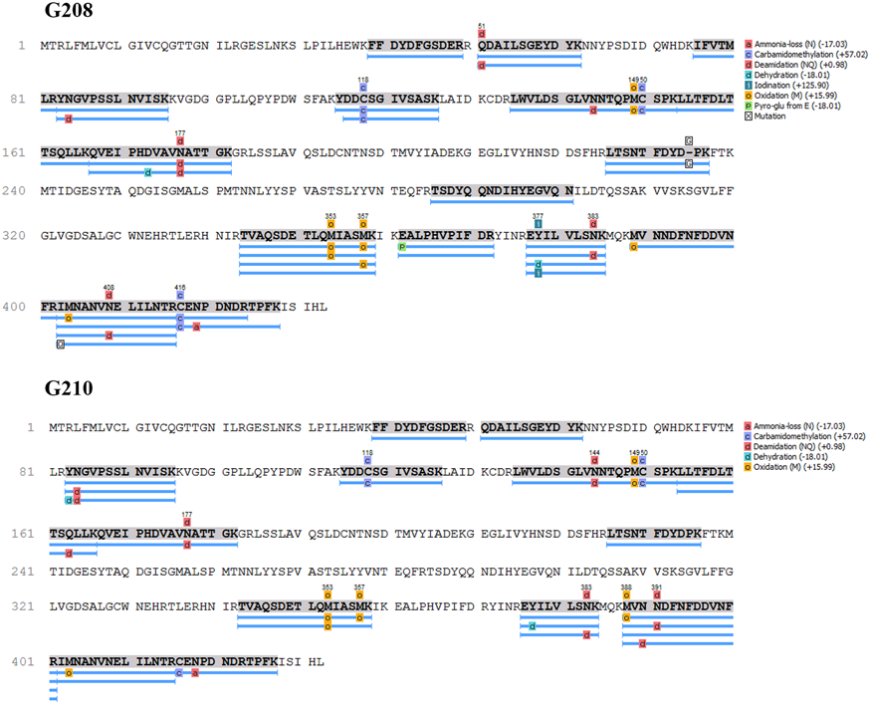
Summary of fully annotated matched peptides between the MRJP1 and 61 kDa glycoproteins G208 and G210. Blue bars indicate sequence coverage and confirmed identifications.

This identification brought important realization that the major royal jelly protein 1 (MRJP1) precursor harbors three known antimicrobial peptides: Jelleins. Fully annotated matched peptides of the deglycosylated 61kDa protein from G208 and G210 are presented in Fig. 12.

**Fig 13:**
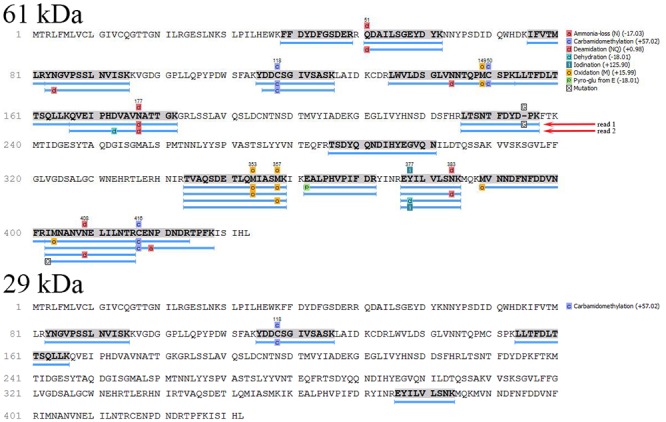
Summary of fully annotated matched peptides between the MRJP1 and 61 kDa and 29 kDa protein. Blue bars indicate sequence coverage and confirmed identifications.

In addition, the most abundant 29 kDa protein co-eluting with the 61kDa MRJP1 band in glps G208 and G177 (Fig. 1a and b) has also been analyzed by MALDI-TOF MS. Mascot database search using the peptide mass fingerprints produced a statistically significant score of 281 (where scores >88 are significant p<0.05) matching major royal jelly protein 1 precuror from *Apis mellifera* (Accession number: gi|58585098). ESI-Q-TOF- MS/MS data showed 98.8% homology with major royal jelly protein 1 precuror from *Apis mellifera* (Accession number: gi|58585098) (Fig. 13), therefore indicating that 29 kDa band was a fragment of the MRJP1 (Table 5). Its presence enhanced cell wall damaging effects of the 61 kDa protein (Fig. 2).

**Table 5:** Summary of ESI-Q-TOF- MS/MS statistical data for 29kDa glycoproteins G177 and G208: Supporting Peptides.

Peptide	Score (%)	-10lgP	Mass	ppm	m/z	RT	Start	End
K.LLTFDLTTSQLLK.Q	99.6	41.83	1491.8549	41.0	746.9653	37.58	154	166
K.YDDCSGIVSASK.L	99.6	40.58	1300.5605	46.2	651.3176	24.58	115	126
R.EYILVLSNK.M	97.7	30.42	1077.6069	37.2	539.8308	32.17	376	384
R.YNGVPSSLNVISK.K	86.2	24.94	1376.7300	33.1	689.3951	30.05	83	95

In contrast, MALDI-TOF sequence data of glp G207 and its 27 kDa fragment excised from the SDS gels (Fig. 1) showed their identity with the Major Royal Jelly Protein 2, MRJP2 of *Apis mellifera* (Table 6). Mascot score of 325 (>55) indicates identity or extensive homology (p<0.05). MRJP2 does not contain antimicrobial Jelleins in its sequence [29]. This could explain the protein agglutinating activity but the low growth inhibitory activity observed in our well diffusion assays.

**Table 6:** Summary of MALDI-TOF-MS statistical data for 58.5 kDa glycoproteins G207.

Accession number	Protein ID	Mr (kDa)	MASCOT score	Matches	Sequences
gi|58585108	MRJP2	51.441	325	6 (2)	5 (2)

## Discussion

The unsuspected finding of this study was the identification of honey glycoproteins as active principal molecules that caused agglutination and a rapid, concentration-depended bactericidal effect on both Gram-negative *E*. *coli* and Gram positive *B*. *subtilis*. The presence of high mannose- type of oligosaccharides in honey glycoproteins allowed their selective isolation using resin-immobilized Concavalin A. Subsequently, we have demonstrated that only the high mannose-type glycoproteins retained by ConA-column showed growth inhibitory and bactericidal activities, while flow-through proteins devoid of mannose-rich glycans were unable to inhibit bacterial growth, reduce bacterial viability or influence bacterial cell shape. These results indicated that the high-mannose structures have a significant role in the antibacterial activity of isolated honey glycoproteins.

Due to the presence of carbohydrate moiety, glps displayed lectin-like activity, agglutinating both Gram-positive *B*. *subtilis* and Gram-negative *E*. *coli*. Agglutinating specificity of honey glps was similar to that of ConA. Both, Glps and ConA had much lower lower reactivity with murine red blood cells than phytohemagglutinin (PHA), but efficiently agglutinated both *E*. *coli* and *B*. *subtilis* cells. This supports reports indicating that ConA binds mannose receptors on *B*. *subtilis* cell wall peptidoglycans [25] and on *E*. *coli* cell envelope [26] with high affinity, but does not recognize branched, complex-type N-glycans, containing galactose on erythrocyte membranes [27]. The binding specificity of ConA and glps allowed the dissociation of agglutinating from hemagglutinating activities. Despite their lack of hemolytic activity, glps increased erythrocyte membrane permeability, affecting osmotic regulation in rat RBCs. This was evident by the appearance of crenated, shrank echinocytes with compromised membranes that showed protrusions and blebs.

Honey glps caused even more severe membrane damage to bacterial cells than to erythrocytes as observed by light and SEM microscopy. The severity of these damages was dependent on glps concentrations, exposure time and the growth phase of bacterium (lag phase versus log phase). At sub-inhibitory glps concentrations, bacterial cells were inhibited in their division and formed long filaments. At bactericidal concentrations, the predominant morphological form was spheroplast that was prone to lysis. While light and SEM microscopy provided evidence that glps caused disruption of the cell wall integrity, the time-kill assays, conducted in parallel to microscopic analysis, showed that the glps-induced damages to the cell wall were lethal. At bactericidal concentrations, glps were able to reduce bacterial counts by >5-log_10_ within 15 min incubation (one generation time).

Taken together, these results indicated that honey glps operated via two distinct functionalities: the high-mannose structure that selectively targets bacterial cells resulting in agglutination, and a less specific membrane permeabilization as observed in both bacterial cells and erythrocytes.

We have recently identified bacterial cell wall as the cellular target for honey antibacterial compounds, however the chemical nature of these compounds remained unknown [17]. The morphological changes and time-kill kinetics of the glps mirror/matched the effects of full honey from which glps were isolated. Glps and full honey both induced phenotype changes in a dose-dependent manner and both caused a rapid, >5log_10_ reduction of viable cell counts during one generation time [17]. These correlations indicate that glps are the active compounds responsible for producing antibacterial effects of honey.

This discovery compelled us to identify which component of glp fraction was relevant to bactericidal activity. The predominate proteins bands on SDS-PAGE were extracted and sequenced. MALDI-TOF and electrospray quadrupole time of flight mass spectrometry, ESI-Q-TOF- MS/MS of the 61 kDa protein band observed in glycoproteins G177, G208 and G210 revealed its identity with the major royal jelly protein 1 precursor (*Apis mellifera*, accession number: gi 58585098) with the confidence greater than 99.2% and 95%, respectively. This identification lead to the important realization that the major royal jelly protein 1 (MRJP1) precursor harbors three known antimicrobial peptides: Jelleins. Previous studies have found that the MRJP1 protein is glycosylated with a high mannose-type structure consisting of Man (9 to approximately 4) GlcNAc2 [28]. The structure and properties of MRJP1 support the observed activity of the glps. The lectin activity and agglutination result from the high mannose glycans in the MRJP1while the presence of Jelleins antimicrobial peptide could explain cell wall damage and cell lysis of both *E*. *coli* and *B*. *subtilis*.

This putative relationship between MRJP1 structure and its activity prompted the investigation into whether agglutination and bactericidal activity occur independently of one another or if glycosylation is an essential prerequisite for antibacterial activity. Deglycosylation of two high-mannose glycoproteins, G207 and G208, had drastically different effect on their antibacterial activities. Deglycosylation of G207 resulted in dramatic loss of antibacterial activity while deglycosylated G208 retained its antibacterial activity. Sequencing of the main 58.5 kDa protein in G207 by MALDI-TOF identified the predominate protein as the major royal jelly protein-2 precursor (MRJP1: *Apis mellifera*, accession number gi|58585108). Unlike MRJP1, MRJP2 does not contain antimicrobial peptides in its sequence [29]. Therefore the presence of Jelleins is likely responsible for the antibacterial activity of G208 and other honeys containing MRJP1.

Membrane disruption and permeabilization has been shown to be a common property of many AMPs including nisin, temporins, cecropins, magainins, mellitins and defensins [30, 31]. The main events involved in bacterial killing by AMPs include attachment to the bacterial membrane, insertion and membrane permeabilization via formation of transmembrane pores and micellarization or dissolution of the membrane [30, 31]. Several models have been proposed to provide a mechanistic view of how the transmembrane pores are formed and lead to the membrane lysis: the barrel-stave model [32], the lytic, detergent-like mechanism represented by the “carpet” model [33] and most recently, the barnacle model [34]. Interestingly, recently discovered AMP hydramacin-1 isolated from *Hydra*, also displayed action with double functionality including bacterial agglutination and membrane disruptions in both Gram-negative and Gram positive bacteria [34].

Macins have a surface of hydrophobic amino acids, and exert their antibacterial action by electrostatic and hydrophobic interactions with negatively charged lipids on the membranes of two neighboring bacterial cells. These interactions lead to the formation of huge bacterial aggregates. In addition to agglutination, neuromacins possesses a pore-forming ability, rapidly permeabilizing membranes of *B*. *megaterium* [35]. The mode of action utilized by Jelleins has not been described and requires further study.

Antimicrobial Jelleins were originally isolated from the royal jelly MRJP1, and were suspected to be produced by the processing of MRJP1 [36, 37]. Research conducted on synthetic Jelleins showed their broad spectrum of activity against Gram-positive and Gram-negative bacteria and against *C*. *albicans* [36]. The MICs of synthetic Jelleins varied between 2.5 μg/ml against *E*. *coli* (CCT 1371) and 15 μg/ml against *S*. *saprophyticus*, and these values are in agreement with the MIC values of glps obtained in this study. The MRJP 1 is protein of bee-origin therefore it is a ubiquitous component of all types of honeys. In fact, honey proteomic studies showed that the proteins of the MRJPs family constitute more than 90% of all honey proteins [38–42]. The MRJP1 proteins have been implicated in several cellular functions [43–45] but had not previously been linked to a role in the destruction of the bacterial cell wall and antibacterial effects of honey. Our results suggest that the MRJP 1 as a source of Jelleins is a disease defense protein and an inherent part of the mechanism of honey bactericidal action.

Here, the connection has been made between bactericidal actions of honey glps containing MRJP1. Our future plans include a study on the mechanism of antibacterial action MRJP1.
